# Effects of tobacco consumption on oral health: Knowledge and Awareness among dental patients and their attitude towards tobacco cessation and counseling in North Maharashtra, India

**DOI:** 10.6026/973206300214329

**Published:** 2025-12-15

**Authors:** Bharath Shivkumar, Jagdishchandra Vathar, Shwetha D, Rajesh Ijalkar, Savreen Kaur, Rini Chacko

**Affiliations:** 1 Department of Public Health Dentistry, Government Dental College and Hospital, Jalgaon, Maharashtra, India; 2 Department of Public Health Dentistry, Government Dental College and Hospital, Chatrapati Sambhajinagar, Maharashtra, India; 3 Orthodontics and Dentofacial Orthopedics, HKES's Nijalingappa Institute of Dental College and Hospital, Kalburgi, Karnataka, India; 4 Department of Pediatric and Preventive Dentistry, Government Dental college and Hospital, Jalgaon, Maharashtra, India; 5 Department of Pediatric and Preventive Dentistry, SGT university, Gurgaon, Haryana, India

**Keywords:** Tobacco chewing, smoking, knowledge, attitude, awareness

## Abstract

India is the fourth-highest tobacco consumer in the world. Therefore, it is of interest to assess knowledge, awareness, attitude and practice of tobacco consumption among dental patients. Hence, a total of 414 dental patients with mean age 38.9±2.5 years were enrolled. The knowledge (p=0.04) and attitude (p=0.02) were significantly associated with education whereas, attitude towards quitting tobacco consumption was associated with age (p=0.05). Thus, participants had moderate knowledge and favorable attitude; proper tobacco cessation counseling will help in quitting the tobacco consumption.

## Background:

Among both rural and urban populations in developing nations, tobacco is one of the most infamous narcotic substances that are abused; this trend may also be seen in countries with advanced economies [1]. Individual behavior has a significant influence on chronic diseases, which can be avoided by altering people's knowledge, attitudes, beliefs, and social surroundings [2]. Globally, a steady increase in the rate of consumption of tobacco products and the number of smokers in the past decade has been reported [3, 4]. Numerous epidemiological and scientific studies have unequivocally shown that cigarette smoking increases the risk of developing degenerative diseases like lung cancer and cardiovascular disorders [5]. Thus, the Framework Convention on Tobacco Control (FCTC) guidelines (WHO-2019) is based on the World Health Organization's (WHO) aggressive global public education efforts about the risks of tobacco use [6]. Smoking status, and consequently smoking-related morbidity and mortality, are strongly correlated with socioeconomic level (SES) [7]. Despite efforts to reduce tobacco use, the rate of smoking prevalence decline has not been consistent among countries and regions, nor has it been consistent across groups with varying socioeconomic position [7]. It must be emphasized that we can anticipate a future expansion of social disparities in health unless steps are taken to decrease the high prevalence of smoking among lower SES groups [8, 9]. Smoking is also detrimental to oral health, causing unaesthetic tooth staining, bad breath, periodontal diseases, impaired healing of wounds, increased risk of dental implant failure, precancerous conditions and oral cancer [10]. Smokeless tobacco is a general phrase that encompasses a variety of items, such as Swedish-style snus, gutkha, zarda, toombak, betel quid, chewing tobacco, and dry and moist snuff [10]. In addition to containing a variety of 4000 chemicals, including over 30 recognized carcinogens, these items are typically produced from tobacco, nicotine, sweeteners, abrasives, salts, and chemicals [11]. Smokeless tobacco delivers 3-4 times more nicotine than smoked tobacco. The amount of nicotine in 8-10 chews/dips per day is equivalent to 30-40 cigarettes per day [11]. WHO's Framework Convention on Tobacco Control (FCTC) and MPOWER have raised awareness of tobacco cessation (TCs) over the world [12]. Although a number of top national institutes, professional dental bodies, the National Tobacco Control Program (NTCP), the National and three Regional Quitlines, and MCessation have made quitting easier, the percentage of former users has been extremely low-less than 2% at the population level [12]. Therefore, it is of interest to learn more about tobacco consumers' sociodemographic condition, awareness, knowledge regarding the ill effects of tobacco abuse, their attitude towards quitting the habit and tobacco cessation counselling in the north Maharashtra region.

## Methodology:

This was a cross-sectional study conducted among the Dental patients visiting Government Dental College and Hospital (GDCH), Jalgaon, Maharashtra. Study was conducted during the month of November 2024 to March 2025. According to Global Adult Tobacco Survey 2016-17, considering the prevalence of tobacco consumption as 28.6%; with absolute precision of 5% and power of 85%, sample size was calculated using Statcalc - Epi Info - Center for Disease Control (CDC) and it came to 378 and is rounded up to 400 [12].

## Inclusion criteria:

Dental patients who consume tobacco were selected by systematic random sampling after taking written informed consent.

## Exclusion criteria:

Dental patients who were not willing to participate in the study.

## Detailed questionnaire regarding knowledge, attitude and practice:

[1] The initial part of the questionnaire contained general socio-demographic details of the participant.

[2] It consists of total ten questions to assess knowledge and awareness about tobacco consumption effects. A Score was given to each question and then participants were classified as having below average knowledge with score with score <6 and above average knowledge with score ≥6.

[3] To identify the perception and attitude of study subjects towards tobacco use, four questions were asked. A score was allotted to each question and was considered as not favorable attitude with score <2 and favorable attitude was with score ≥2.

## Statistical analysis:

Collected data were entered in MS Excel and analyzed using IBM-SPSS® Statistics-Version 21 [USA: IBM Corp.]. Descriptive statistics was applied for the frequency distribution and percentage of dental patients. Chi-square test for the association between the study variables, knowledge, and attitude questions.

## Results:

A total 414 participants were included in this study. Mean age of study subjects was 38.9±2.5 years ranging from 20 yrs to 60 yrs. Out of these participants, 286 (69.1%) are male and 128(30.9%) are female. 249 (60.1%) of the subjects belong to below poverty line (BPL) and 165 (39.8%) belong to above poverty line (APL). Among education qualification 382 (92.3%) were undergraduates and 32 (7.7%) were postgraduates. When the knowledge of participants was assessed for tobacco consumption, it was found that majority were aware of the addictive nature of tobacco, its harmful effects to self and others. The major source of information for these participants was mass media i.e. 260 patients (62.8%). Table 1 (see PDF), shows various socio-demographic factors affecting knowledge. It shows education of the patients was significantly associated with knowledge and awareness about ill-effects of tobacco consumption (p=0.04) while age and socio-economic status were not significantly associated. Also, age (p=0.05) and education (p=0.002) were significantly associated with attitude towards quitting tobacco consumption.

## Discussion:

Tobacco consumption is widespread in India. In our study, 78.3% patients had good knowledge about ill effects of tobacco consumption effects whereas 92.3% have favorable attitude towards tobacco quitting [13]. Among study subjects, 67.7% were tobacco chewers and 26.4% were smokers and 6.9% consumed tobacco in both smoked and chewable form [13]. Mean age of initiation of tobacco consumption was 38.9±2.5 years. The prevalence rate of tobacco use in this present study shows results similar to those of other studies [14]. Sinha *et al.* had reported very high tobacco use i.e. 74.15% in a village in Bihar among males [15]. Social influence affects public health, established fact that People eventually switch from being smokeless to smoking, which will eventually endanger and strain the country's health and economy [16, 17]. Sociodemographic status had no statistically significant relation to awareness, knowledge (P=0.28) and attitude (p=0.15) of the participants, which was in contrast to studies done by Nagrath *et al.* [18] and Barik *et al.* [19]. Understanding the different interventions, mode of treatments and characteristics of nicotine intoxication is crucial in order to help people break the habit through tobacco cessation counselling.

## Conclusion:

The study suggests though the knowledge of the participants toward tobacco consumption is adequate, tobacco consumption habit is higher. This gap can be reduced by proper counseling of the dental patients. There is a need for dental practitioners and health workers to routinely educate all the patients they come in contact with, laying more emphasis on the rare complications of tobacco use. Dentists are in a strategic position and can play a crucial role in creating awareness and provide smoking cessation counselling, effectively. The media can be a great tool for wider coverage and the health professionals should take advantage of that. The use of multiple regulatory strategies should be employed to reduce the overall adverse health impact of tobacco.

## Funding:

No funding sources.

## Ethical approval:

The study was approved by the Institutional Ethics Committee.
